# Immunosuppressive Activity of Daphnetin, One of Coumarin Derivatives, Is Mediated through Suppression of NF-κB and NFAT Signaling Pathways in Mouse T Cells

**DOI:** 10.1371/journal.pone.0096502

**Published:** 2014-05-06

**Authors:** Bocui Song, Zhenning Wang, Yan Liu, Sisi Xu, Guoren Huang, Ying Xiong, Shuang Zhang, Linli Xu, Xuming Deng, Shuang Guan

**Affiliations:** 1 Key Laboratory of Zoonosis, Ministry of Education, College of Veterinary Medicine, Jilin University, Changchun, People’s Republic of China; 2 Department of Food Quality and Safety, College of Light Industry Economics and Management, Jilin University, Changchun, People’s Republic of China; Emory University, United States of Amreica

## Abstract

Daphnetin, a plant-derived dihydroxylated derivative of coumarin, is an effective compound extracted from a plant called Daphne Korean Nakai. Coumarin derivates were known for their antithrombotic, anti-inflammatory, and antioxidant activities. The present study was aimed to determine the immunosuppressive effects and the underlying mechanisms of daphnetin on concanavalin A (ConA) induced T lymphocytes in mice. We showed that, *in vitro*, daphnetin suppressed ConA-induced splenocyte proliferation, influenced production of the cytokines and inhibited cell cycle progression through the G0/G1 transition. The data also revealed that daphnetin could down-regulate activation of ConA induced NF-κB and NFAT signal transduction pathways in mouse T lymphocyte. *In vivo*, daphnetin treatment significantly inhibited the 2, 4- dinitrofluorobenzene (DNFB) -induced delayed type hypersensitivity (DTH) reactions in mice. Collectively, daphnetin had strong immunosuppressive activity both *in vitro* and *in vivo*, suggesting a potential role for daphnetin as an immunosuppressive agent, and established the groundwork for further research on daphnetin.

## Introduction

Immunosuppressive drugs are used for the treatment of undesirable or abnormal activation of T lymphocytes and the immune system associated with organ transplantation and autoimmune diseases. T lymphocytes play a pivotal role in the pathogenesis of cell-mediated autoimmune diseases and the chronic inflammatory disorders [1], [2], [3]. Activation of T-lymphocytes requires stimulation of T-cell receptors (TCR) and co-stimulatory signals. Ca^2+^ influx is crucial for T cell activation upon antigen stimulation [4]. The alteration in intracellular calcium ([Ca^2+^]i) controls diverse cellular processes. Changes of [Ca^2+^]i can promote translocation of transcription factors from the cytosol to the nucleus. The best known examples are those of NF-κB (nuclear factor kappa -light-chain -enhancer of activated B cells) and NFAT (nuclear factor of activated T-cells) [5], [6]. The calcineurin–NFAT signaling pathway is activated by an increase of the cytosolic Ca^2+^ level. Ca^2+^ binds to the catalytic subunit of calcineurin through calmodulin, which leads to dephosphorylation of cytosolic NFAT and promotion of its nuclear translocation [7], [8]. NF-κB is also activated either by Ca^2+^/calmodulin dependent protein kinase (CaMK II) [9], [10]. NFAT and NF-κB translocates to the nucleus and turns on transcription of specific genes, generally related to inflammatory or immune responses, cell survival responses or cell proliferation.

In alternative medicine, plants have long been used to treat a wide range of pathologies, such as cancers, autoimmune diseases, inflammatory diseases and asthma. Daphnetin (7, 8-dihydroxycoumarin), one of coumarin derivatives, is an effective compound extracted from a plant called Daphne Korean Nakai, with a molecular weight of 178 [11], [12]. It has been clinically used in the treatment of coagulation disorders, rheumatoid arthritis, lumbago and to reduce fever [13], [14], [15]. Furthermore, the daphnetin displays a significant anti-cancer effect and inhibits kinase activity *in vitro* [16], [17]. The daphnetin is also capable of producing notable anti-proliferative activity among different malignant cell lines [18]. These findings indicate that daphnetin has the potential immunosuppressive function through its effect on cells. However, the underlying immunosuppressive mechanisms of daphnetin are still largely unknown. So the immunosuppressive activity of daphnetin is needed to be investigated further investigate. In this study, we investigated the *in vitro* and *in vivo* immunosuppressive effect of daphnetin on BALB/c mice T lymphocytes, and explored the potential mechanism underlying this effect. We found that daphnetin significantly inhibited ConA-stimulated splenocyte proliferation, cytokine production and cell cycles *in vitro*. The data also showed that daphnetin exhibited an immunosuppressive effect on T cell activation through the Ca^2+^-calcineurin-NFAT and NF-κB pathways. Moreover, we examined the effect of daphnetin on DNFB-induced T cell subsets, ear swelling and inflammatory cell infiltration. Our results suggested that daphnetin had protective effects of DTH induced by DNFB. The study of its immunosuppressive mechanisms is bound to provide some useful information on its promising therapeutic application.

## Materials and Methods

### Ethics statement

All animal studied were conducted according to the experimental practices and standards approved by the Animal Welfare and Research Ethics Committee at Jilin University (Approval ID: 20110520-3). Animal experiments were carried out on 18∼22 g male BALB/c mice.

### Chemicals

Daphnetin (purity>98%, catalog number:110900) was obtained from the National Institute for the Control of Pharmaceutical and Biological Products (Beijing, China). Daphnetin was diluted in culture medium with 0.1% dimethylsulfoxide (DMSO, Sigma, St. Louis, MO, USA) and then sterile-filtered before used. All controls and samples contained the same concentrations of DMSO.

Concanavalin A (ConA) and 3-(4, 5-dimethylthiazol-2-y1)-2, 5-diphenyltetrazolium bromide (MTT) were purchased from Sigma Chemical Co. (St. Louis, MO, USA). The mAbs against mouse CD3, CD4, CD8, IFN-γ, IL-2, IL-4 and IL-6 ELISA kits were purchased from Biolegend (California, USA). RPMI-1640 medium was purchased from HyClone (Logan, UT), and fetal bovine serum (FBS) was obtained from Invitrogen-Gibco (Grand Island, NY). Primary antibodies used for Western analysis Calmodulin, Calcineurin (CaN), Ca^2+^/calmodulin-dependent kinase II (CaMKII), P-CaMKII, NF-κB, which were purchased from Cell Signaling (beverly, MA), NFAT2 antibodies was purchased from Abcam (Cambridge, MA), β-actin was obtained from Tianjin Sungene Biotech Co., Ltd. Goat anti-mouse IgG, Goat anti-mouse FITC, Peroxidase-conjugated AffiniPure goat anti-mouse IgG (H + L) were purchased from Protein Tech Group, 2, 4-Dinitrofluorobenzene (DNFB) and cyclophosphamide (CTX) were obtained from Sigma-Aldrich (St. Louis, MO).

### Experimental animals

BALB/c male mice, weighing approximately 18 to 22 g, were purchased from Jilin University Experimental Animal Center and acclimatized for 1 week before use. The mice were housed in microisolator cages and received food and water ad libitum. All animal studies were conducted according to the experimental practices and standards approved by the Animal Welfare and Research Ethics Committee at Jilin University.

### Cytotoxicity assay

Splenocytes collected from BALB/c male mice under aseptic conditions were mechanically scraped, and plated at a density of 2×10^6^ cells/mL onto 96-well plates containing 100 µL of RPMI 1640 complete medium. Then the cells were treated with multiple concentrations of daphnetin (0–64 µg/mL). The plate was incubated at 37°C with 5% CO_2_. After that, MTT was added at a final concentration of 5 mg/mL and incubated for 4 h at 37°C with 5% CO_2_. Cell-free supernatants were then removed and and resolved with 150 µL/well DMSO. The optical density was measured at 570 nm on a microplate reader. The experiment was repeated three times.

### ConA induced splenocyte proliferation assay *in vitro*

Splenocytes proliferation assays were performed in 96-well round-bottom plates (Costar, Cambridge, MA) in a total volume of 0.2 mL RPMI 1640. Briefly, BALB/c splenocytes suspension (2 × 10^6^/mL) cells well were stimulated with ConA (5 µg/mL) and different concentrations of daphnetin (4, 8, 16 µg/mL) in a 96-well flat-bottomed plate. The cell cultures were then incubated for 44 h at 37°C in a humidified 5% CO2 incubator. After 44 h, 20 µL of MTT solution (5 mg/mL) was added to each well and incubated for 4 h. Then the culture medium was removed and 150 µL of DMSO was added to lyse the cells and the plate was read at 570 nm. The experiment was repeated three times.

### Analysis of T lymphocyte cycle by flow cytometry

Splenocytes were seeded in 24-well plates (2×10^6^ cell s/well) and then treated with different concentrations of daphnetin (4, 8, 16 µg/mL) plus Con A (5 µg/mL) and ConA alone for 24 h, then washed twice with ice-cold PBS and fixed by 70% ethanol at −20°C for at least 24 h. Cells were washed twice with ice-cold PBS and stained with 50 mg/ml of propidum iodide (PI) in the presence of 100 mg/ml RNase A for 0.5 h. Data acquisition was done by flow cytometry (Becton &Dickinson, U.S.A.). At least 10000 cells per sample were collected and analyzed by using the Cell Fit Cell analysis program.

### Determination of cytokine levels *in vitro*

Splenocytes were plated onto 24 -well plates (2×10^6^ cell s/well) and incubated in the presence of either ConA alone (5 µg/mL) or ConA plus daphnetin (4, 8, 16 µg/mL) for 24 h at 37°C with 5% CO2. Cell-free supernatants were collected and stored at −20°C until assayed for cytokine concentrations. The productions of IFN-γ, IL-2, IL-4 and IL-6 in ConA-stimulated splenocytes were measured by commercial ELISA kits.

### T cell purifications

T cells were purified from whole splenocyte preparations using the Pan T Cell Isolation Kit according to the manufacturer’s instructions (Miltenyi Biotec, Bergisch Gladbach, Germany). Briefly, splenocytes were collected and a single cell suspension was generated in MACS buffer (PBS, 0.5% BSA and 2 mM EDTA). Cells were then incubated with antibody cocktail and magnetic beads that allow for negative selection of T cells in the presence of a magnetic column. The purity of mouse CD3 T cells was consistently more than 95%.

### Determination of [Ca^2+^]i

The intracellular free Ca^2+^ concentration ([Ca^2+^]i) of mouse CD3 T cells was measured by using Fluo-3-AM staining. In brief, mouse CD3 T cells (2×10^6^ cells/mL) were plated onto 6-well plates and pretreated with daphnetin (4, 8, 16 µg/mL) plus Con A (5 µg/mL) and ConA alone for 0.5 h. Then the cells were incubated with the fluorescent calcium indicator Fluo-3/AM (20 µM) for 30 min in the presence of 1 µM pluronic acid F-127 at 37°C. The cells were then washed 3× with HBSS buffer to remove nonhydrolyzed Fluo-3/AM. [Ca^2+^]i was calculated using the formula : [Ca^2+^]i = Kd [F-Fmin]/[Fmax-F], where F min and Fmax are the fluorescence levels at zero and saturating ion concentrations, respectively, and Kd for Fluo-3/AM is 400 nM. Fluorescence measurements were performed using a Confocal Laser Scanning Microscope.

### Western Blotting analysis

Protein concentrations were determined by the Bio-Rad protein assay. Mouse CD3 T cells (2×10^6^ cells/mL) in 2 mL of RPMI 1640 complete medium were incubated with 1 mL of daphnetin (4, 8, 16 µg/mL) in 6-well plates at 37°C for 1 h followed by 0.5 h incubation with 1 mL ConA (final concentration 5 µg/mL), resulting in a final well volume of 4 mL per well. After 1 h, cell lysates were subjected to SDS-PAGE and transferred to nitro cellulose membrane, and Western blotting was performed with the appropriate antibodies. The proteins were visualized by enhanced chemiluminescence (ECL).

### Immunofluorescense

The nuclear distribution of NFAT and NF-κB were measured using immunofluorescense. Mouse CD3 T cells (2×10^6^ cells/mL) in 2 mL of RPMI 1640 complete medium were incubated with 1 mL daphnetin (4, 8, 16 µg/mL) in 6-well plates at 37°C for 1 h followed by 0.5 h incubation with 1 mL of ConA (5 µg/mL), resulting in a final well volume of 4 mL per well. After over-night incubation in anti-NFAT2 antibody (1∶350) or anti-NF-κB antibody (1∶100), the cells were washed twice and incubated with Goat anti-mouse FITC-linked secondary for 1 h. Fluorescence images were visualized using an Olympus Confocal Laser Scanning Biological Microscope and analyzed using Olympus Fluoview Ver. 1.7a viewer software.

### DNFB-induced delayed type hypersensitivity (DTH) response

Six-week-old female BALB/c mice were divided into 6 groups, each consisting of ten mice. On days 1 and 2, BALB/c mice were initially sensitized with 20 µL of 5% DNFB dissolved in acetone–olive oil (4:1) on the shaved abdominal skin of recipients. Beginning on the day of immunization, the immunized mice were administered by intraperitoneal injection with daphnetin (5, 10, 20 mg/kg, in 2% DMSO) or CTX (20 mg/kg in 2% DMSO) for 5 days once daily. The control groups received the same volume of saline in 2% DMSO. After 5 days, the DTH reaction was elicited by smearing 10 µL of 5% DNFB on both sides of the left ear. The DTH reaction was evaluated by the increase in the ear patch weight (8-mm punches) between the left and right ear was measured 24 h after the second challenge.

### Analysis of T cell surface markers by flow cytometry in DTH mice

For evaluation of the percentage and number of CD3, CD4 and CD8 cells, splenocytes were isolated from mice of all groups. T cell surface markers were determined by staining cells with Cy5.5-anti-CD3, FITC-anti-CD4 and PE-anti-CD8. The splenocytes were incubated with the conjugated-antibodies for 0.5 h at 4°C. After incubation, the cells were washed with PBS at 4°C and analysed by flow cytometry. The Cell Quest software (Becton-Dickinson) was used to identify and quantify distinct populations of cells by mean fluorescent intensity (MFI). A minimum of 10,000 cells were analyzed for each sample.

### Histologic Analysis

All the mice were sacrificed and their right ears were removed. Samples were taken 24 h after final DNFB administrations, fixed in 10% buffered formalin, embedded in paraffin, and sectioned for hematoxylin and eosin (H&E) staining.

### Statistical Analysis

All data were presented as mean ± SD. Data analysis used SPSS version 18.0 (SPSS Inc., Chicago, IL, USA). Comparison of more than two groups was made with a one-way analysis of variance ANOVA followed by Dunnett *t* test. Statistical significance was accepted when *P*<0.05 or *P*<0.01.

## Results

### Cytotoxicity and inhibitive activity evaluation of daphnetin

**Figure 1:**
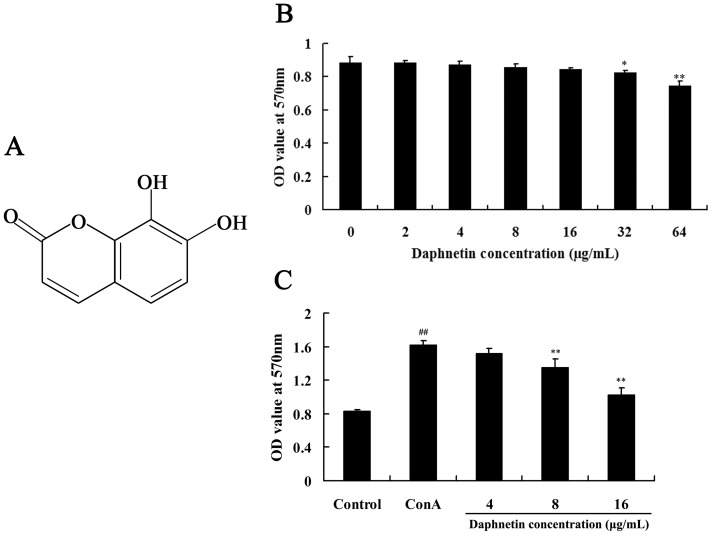
Effect of daphnetin on mouse splenocytes viability and proliferation. (A) Chemical structure of daphnetin used in the study. (B) Effect of daphnetin on the viability of mouse splenocytes. The cells were treated with daphnetin (0–64 µg/mL) for 48 h. The cell viability was determined by MTT assay. (C) Effect of daphnetin on the ConA induced mouse splenocytes proliferation. Splenocytes cultured with fisetin (4, 8, 16 µg/mL) combined with ConA (5 µg/mL) for 24 h. Cell proliferation was assessed by MTT assay. Data are presented as means ± SD of three independent experiments. Significant differences from control group were indicated by *P<0.05 and **P<0.01.

Splenocytes with and without ConA were treated with different doses of daphnetin (4, 8, 16 µg/mL) and measured f or 48 h by MTT method. We demonstrated that daphnetin at concentrations of 16 µg/mL or under had no effect on the viability of splenocytes. At concentrations of 32 µg/mL, daphnetin reduced the cell viability (Figure 1B). The activity of daphnetin was evaluated on mouse splenocytes proliferation induced by ConA *in vitro*. As shown in Figure 1C, a significant increase in the proliferation of splenocytes stimulated by ConA at 5 µg/mL was observed compared to the blank control (*P*<.01). The proliferation index of daphnetin at the concentration 4 µg/mL, 8 µg/mL and 16 µg/mL were comparable to the positive control of ConA used at a concentration of 5 µg/mL. These results indicate that daphnetin strongly suppressed T cell proliferation in a dose-dependent manner. The data suggested that daphnetin had a strong effect on splenocytes proliferation *in vitro*.

### Effects of daphnetin on cell -cycle progression of T cells

**Figure 2:**
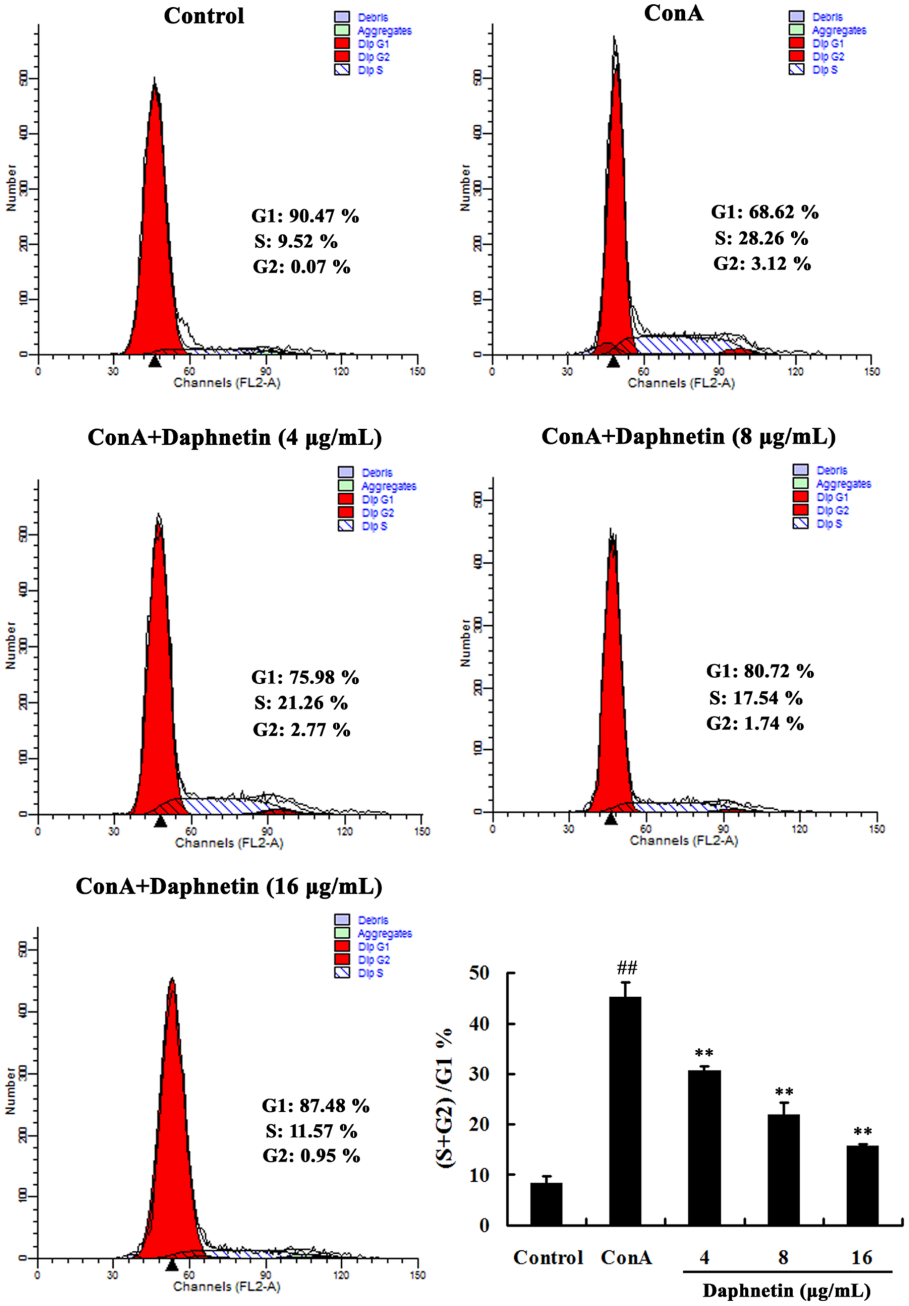
Cell cycle arrest of splenocytes lymphocytes by daphnetin. Cells were harvested and DNA contents were stained with propidium iodide for flow cytometric analysis. The results were from three independent experiments and presented as mean ± SD. ^##^ *P*<0.01 vs. Control group; **P<0.01 vs. ConA group.

The evaluation of cell cycle was confirmed with flow cytometry analysis. The percentage of cells in G0/G1, S, and G2/M phases were calculated with multicycle software as shown in Figure 2, the results indicated that after T lymphocytes were exposed to daphnetin for 24 h, the percentage of cells in G1 stage increased from 76.62%±1.71% to 86.48%±0.30%, the percentage of S stage cells decreased from 20.83%±1.30% to 12.90%±0.50% and the percentage of G2/M stage cells decreased from 2.55%±0.07% to 0.67%±0.22%. Daphnetin blocked DNA synthesis at G1 stage to inhibit mouse T lymphocytes mitosis and cell over-proliferation.

### Influence of daphnetin on Th1 and Th2 cytokines

**Figure 3:**
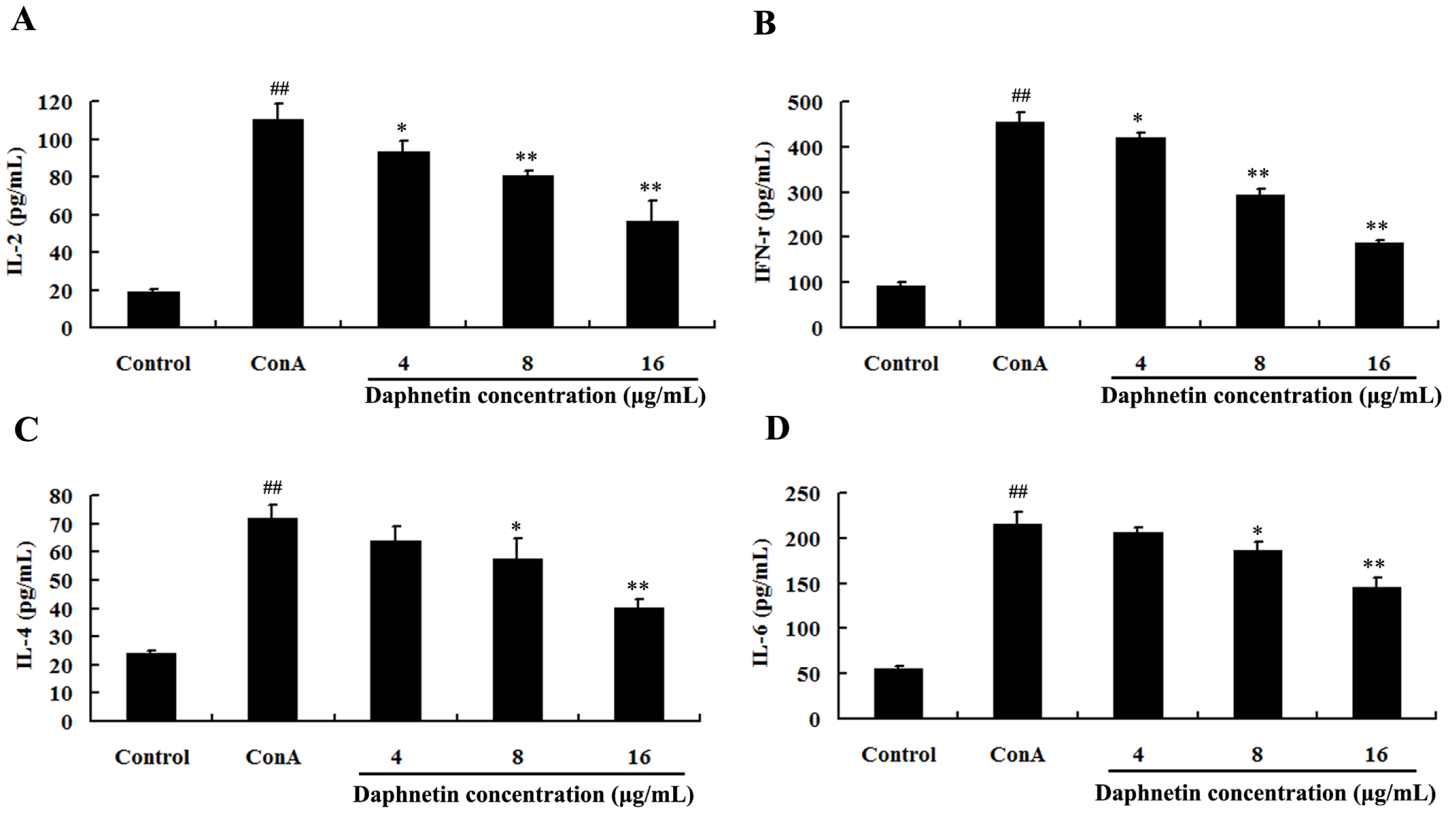
Effect of daphnetin on cytokines secretion in cell culture supernatant. The levels of cytokines IL-2 (A), IFN-γ (B), IL-4 (C) and IL-6 (D) were analyzed after culturing the splenocytes in the presence of different concentration daphnetin and ConA (5 µg/mL) for 24 h. The cytokine levels were quantified by ELISA. The experiments were performed in triplicates and the data were present as means ± SD. ^##^P<0.01 vs. Control group. *P<0.05 or **P<0.01 vs. ConA group.

We used Con A as stimuli to promote cytokine secretion in mouse splenocytes. Then the effects of daphnetin on the production of cytokines IL-2, IFN-γ, IL-4, and IL-6 were examined. In Figure 3, ConA strongly stimulated cytokines production increased rapidly with the incubation time up to 24 h. It was observed by the ELISA method that the IL-2 (Figure 3A) and IFN- γ (Figure 3B) levels were evidently inhibited in groups treated with daphnetin at the dose of 4, 8 and 16 µg/mL. IL-4 (Figure 3C), and IL-6 (Figure 3D) levels in the cell supernatant treated with 8 and 16 µg/mL of Daphnetin significantly decreased compared to those of ConA group.

### Effects of daphnetin on [Ca^2+^]i in T cells

**Figure 4:**
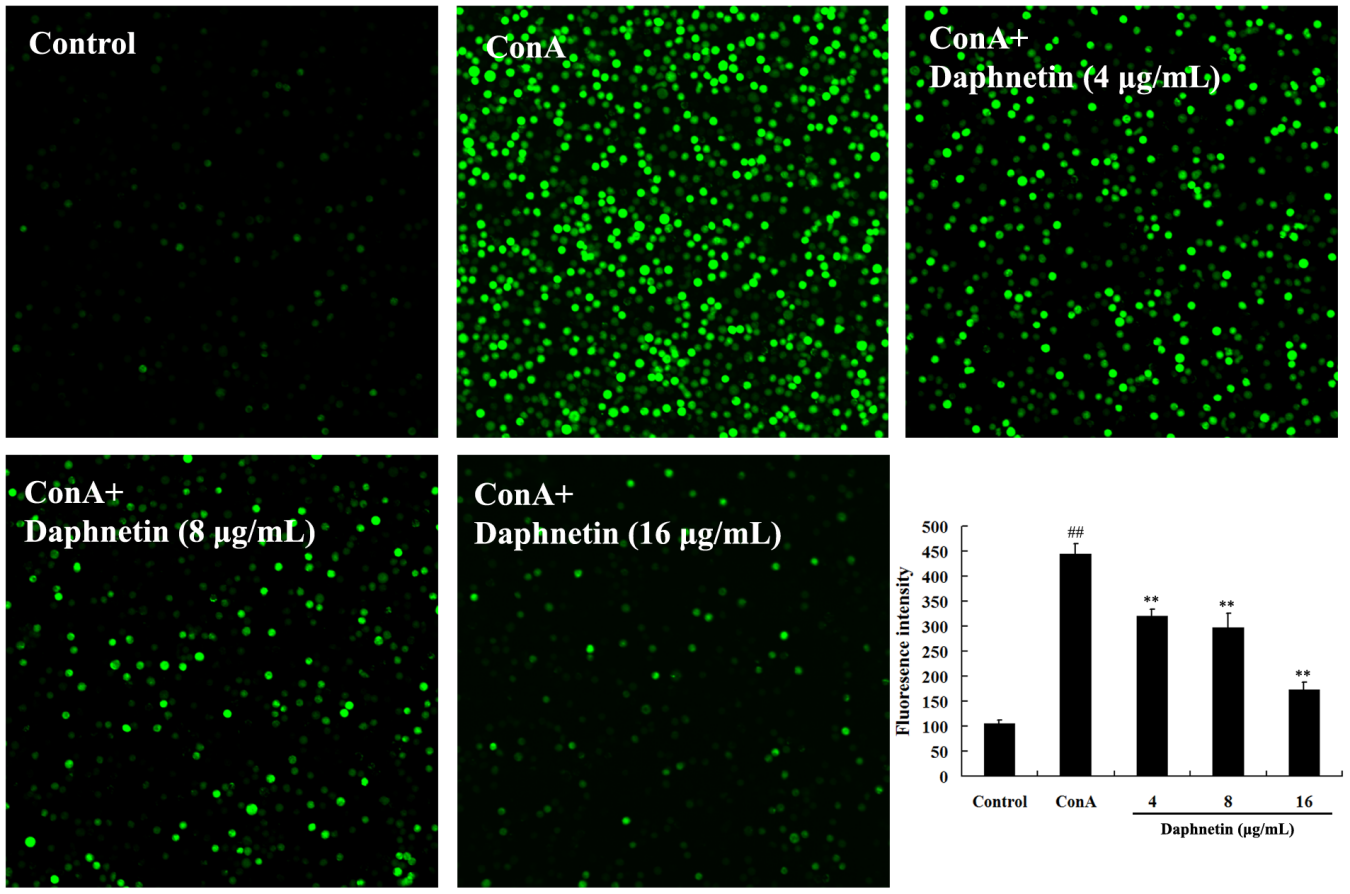
Effects of different concentration of daphnetin on the [Ca^2+^]i in mouse T cells. T Cells were pretreated with 20 µM Fluo-3-AM and incubated in the presence of daphnetin (4, 8, 16 µg/mL) for 30 min at 37°C and measured at 37°C using a Confocal Laser Microscope. The fluorescence intensity in ConA group was increased compared with the control group, and daphnetin could diminish the fluorescence intensity. The results were from three independent experiments and presented as mean ± SD. ^##^ *P*<0.01 vs. Control group; **P<0.01 vs. ConA group.

We used Fluo-3 AM to examine the effects of daphnetin on intracellular Ca^2+^ mobilizations in mouse T cells. In control group, the level of intracellular free Ca^2+^ was the lowest. In ConA group, the level of intracellular Ca^2+^ increased significantly (Figure 4). As the daphnetin concentration at 4, 8 and 16 µg/mL, intracellular free Ca^2+^ fluorescence decreased dramatically. It indicated that daphnetin strongly inhibited the intracellular free Ca^2+^ levels in T cells.

### Repression of calcineurin and phosphor-CaMKII protein expression by daphnetin

**Figure 5:**
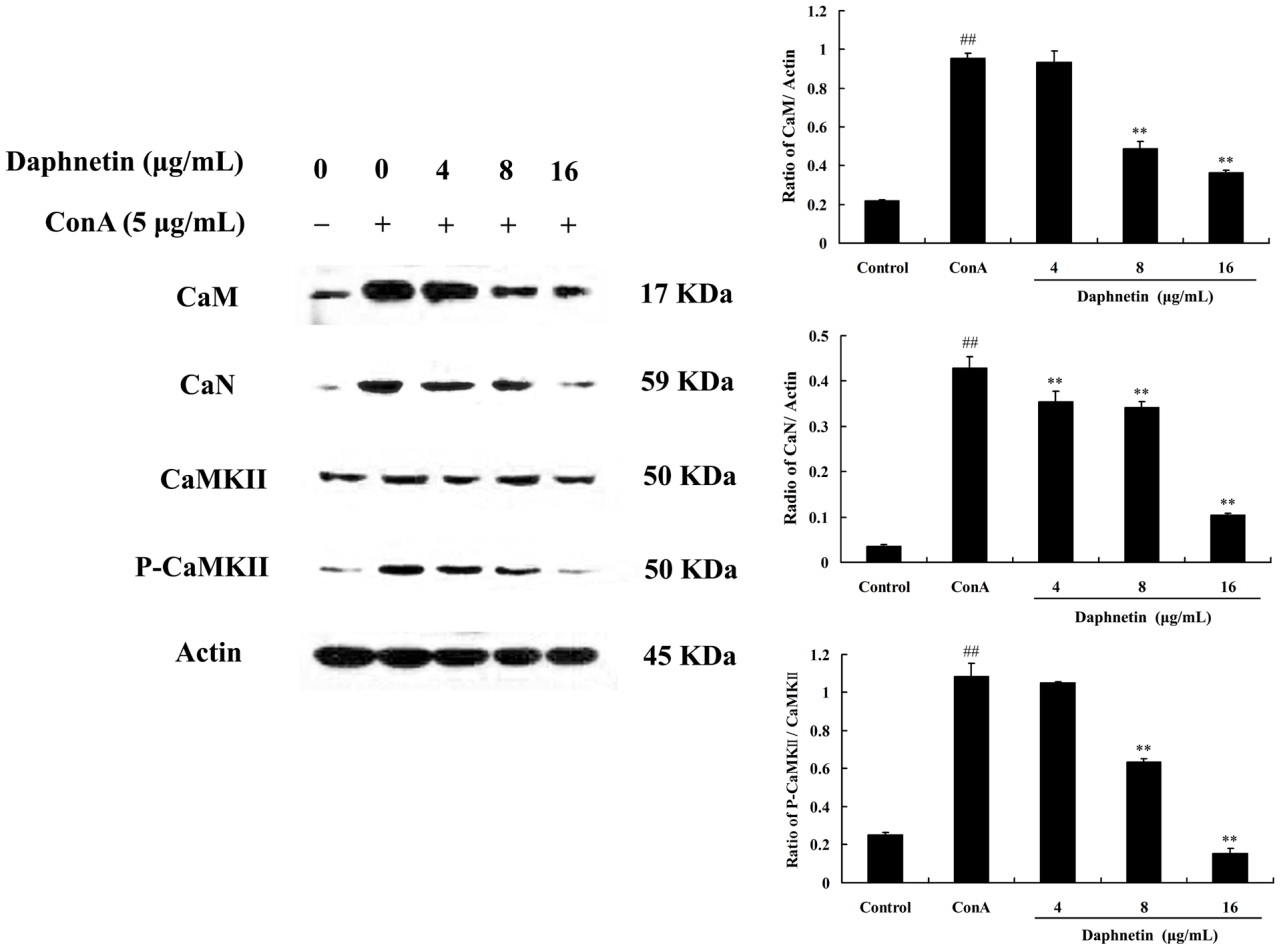
Effect of daphnetin on [Ca^2+^]i -related signaling pathway in mouse T cells. Mouse CD3 T cells were treated with daphnetin (4, 8, 16 µg/mL) for 0.5 h and then stimulated with ConA (5 µg/mL) for 0.5 h. Representative western blots showed protein expression of CaM, CaN, CaMKII and P-CaMKII. Data were presented as means ± SD of three independent experiments. ^##^ *P*<0.01 vs. Control group; **P<0.01 vs. ConA group.

Calmodulin (CaM) is a key regulator of numerous cellular processes and is the predominant intracellular receptor for Ca^2+^ signals. Calcineurin (CaN) is a Ca^2+^-dependent protein phosphatase, often called protein phosphatase-2B (PP-2B), and is known to be involved in some functions of various cells[19], [20]. CaMKII is also an important downstream target of Ca^2+^ signaling pathways [21]. In this study, when T cells were stimulated with ConA, the level of CaM and CaN were increased and daphnetin (4, 8, 16 µg/mL) suppressed the level of CaM and CaN in a dose-dependent manner. We further examined whether the effect of daphnetin on ConA-induced CaMKII by western blot. Western blot analysis shows a significant phosphorylation of CaMKII with ConA stimulation, however, the phosphorylation of CaMKII was inhibited by application of daphnetin at the doses of 8 µg/mL and 16 µg/mL (Figure 5).

### Effects of fisetin on ConA-induced NFAT and NF-κB pathway

**Figure 6:**
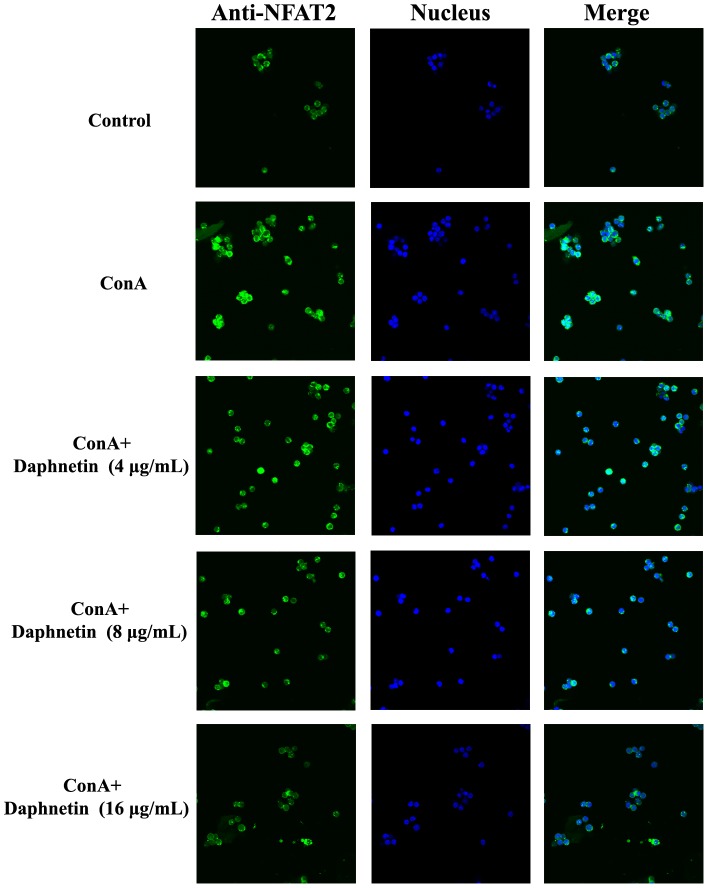
Effect of daphnetin treatment on nuclear translocation of NFAT induced by ConA. Mouse CD3 T cells were pretreated with different concentrations of daphnetin (4, 8, 16 µg/mL) for 1 h and then stimulated with ConA for another 30 min. NFAT localization was analyzed by immunocytochemistry and confocal microscopy.

**Figure 7:**
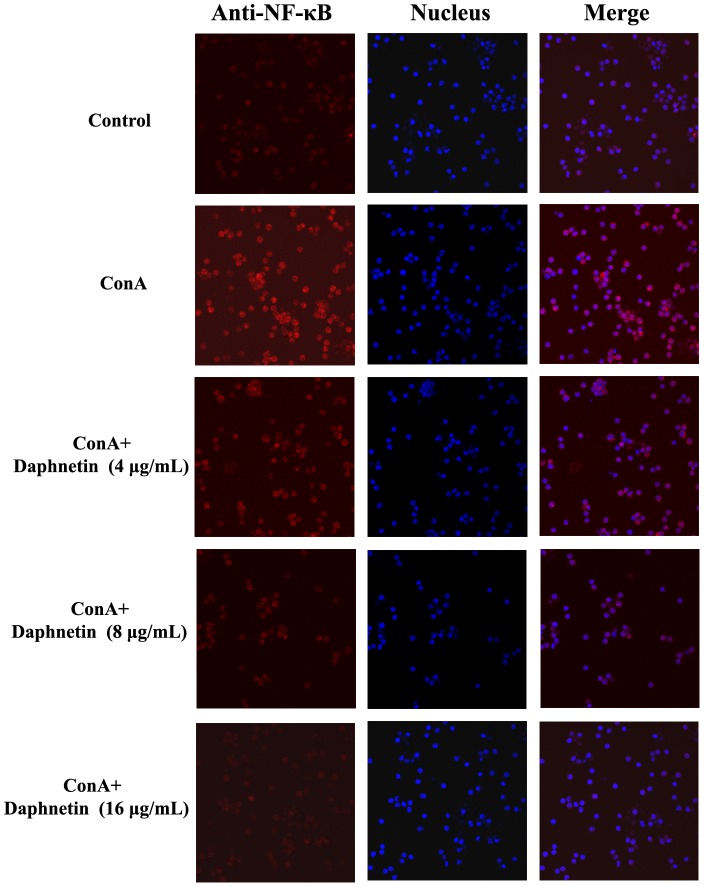
Influence of daphnetin on NF-κB translocation in mouse T cells. Mouse CD3 T cells were treated with 4, 8 and 16 µg/mL of daphnetin for 1 h and then stimulated with ConA for 30 min. Control group: NF-κB was localized to the cytoplasm; ConA group: Cells treated with ConA showed nuclear distribution of NF-κB; Daphnetin group: The localization of NF-κB was mainly cytoplasmic, demonstrating a reduction in the ability to translocate NF-κB. The fluorescence images were recorded with an Olympus Confocal Laser Scanning Biological Microscope.

In order to investigate the mechanism of daphnetin action, we examined its effect on the nuclear translocation of NFAT2 and NF-κB. As shown in Figure 6 and Figure 7, NFAT2 and NF-κB localization were cytosolic in nonstimulated T cells, whereas upon stimulation of mouse CD3^+^ T cells with ConA, NFAT2 and NF-κB translocates rapidly into the nucleus. As expected, this process can be blocked by daphnetin(4, 8, 16 µg/mL).

### Effect of daphnetin on T-cell subsets in DNFB induced DTH reaction in mice

**Figure 8:**
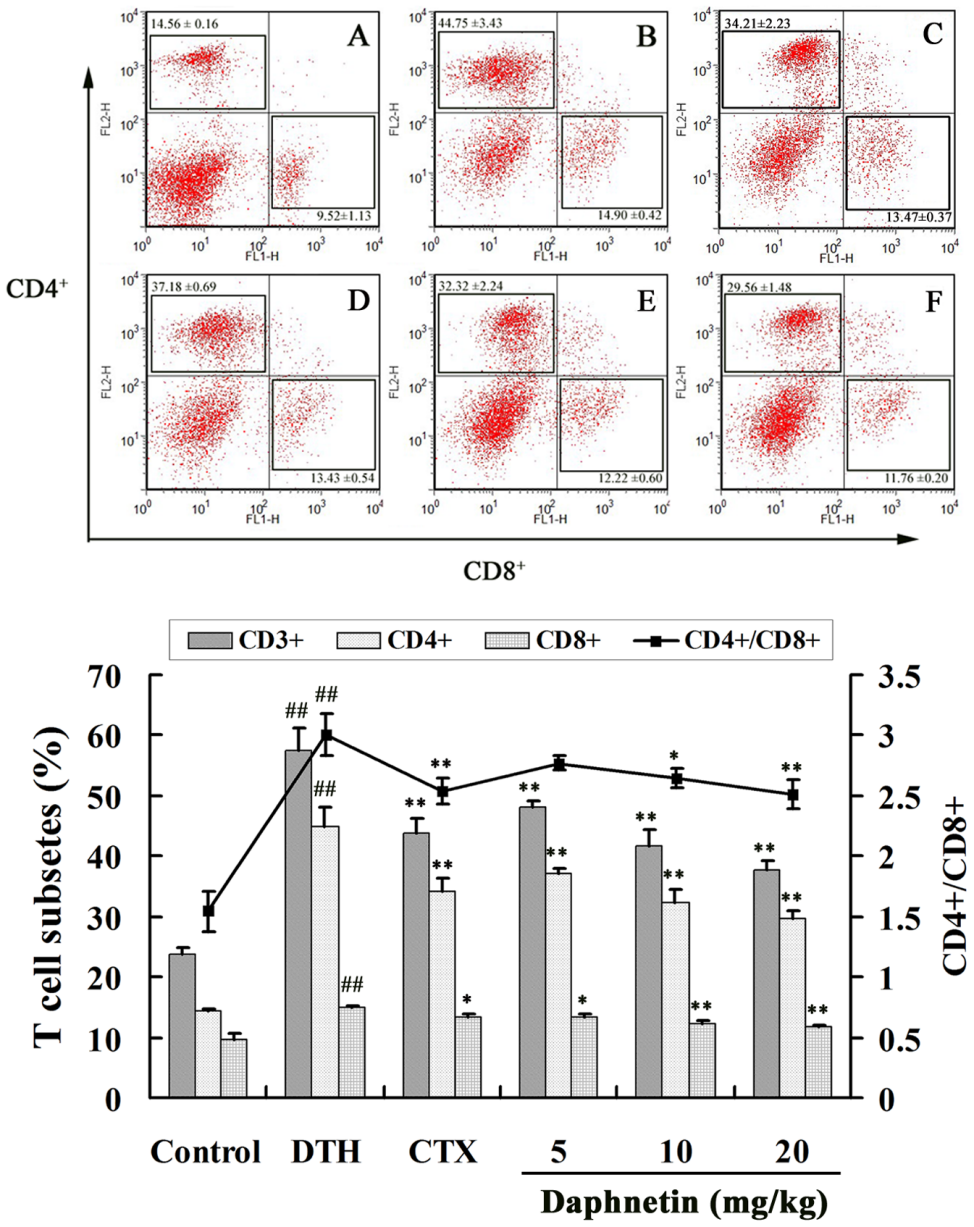
Flowcytometric evaluation of the effect of daphnetin on CD4 and CD8. (A) Control group, (B) DTH group, (C) CTX group, (D) Daphnetin 5 mg/kg group, (E) Daphnetin 10 mg/kg group, (F) Daphnetin 20 mg/kg group. The results were from three independent experiments and presented as mean ± SD. ^##^ *P*<0.01 vs. Control group. *P<0.05 or **P<0.01 vs. ConA group.

Delayed-type hypersensitivity (DTH) is a classic T cell-mediated immune reaction and plays an essential role in the pathogenesis [22]. The effect of daphnetin on CD3^+^, CD4^+^ and CD8^+^ counts was studied using flow cytometric analysis in DTH mice. CD4^+^ and CD8^+^ levels and the ratio of CD4^+^/CD8^+^ were higher in the DTH mice (Figure 8B) than that in the control mice (Figure 8A). However, we found a significant decreased of the CD4^+^ and CD8^+^ T-cell populations and the ratio of CD4^+^/CD8^+^ after daphnetin plus ConA treatment (Figure 8D, 8E and 8F). It meant that daphnetin could inhibit the activation of T lymphocytes in a dose-dependent manner.

### Effect of daphnetin on DNFB-induced ear swelling response

**Figure 9:**
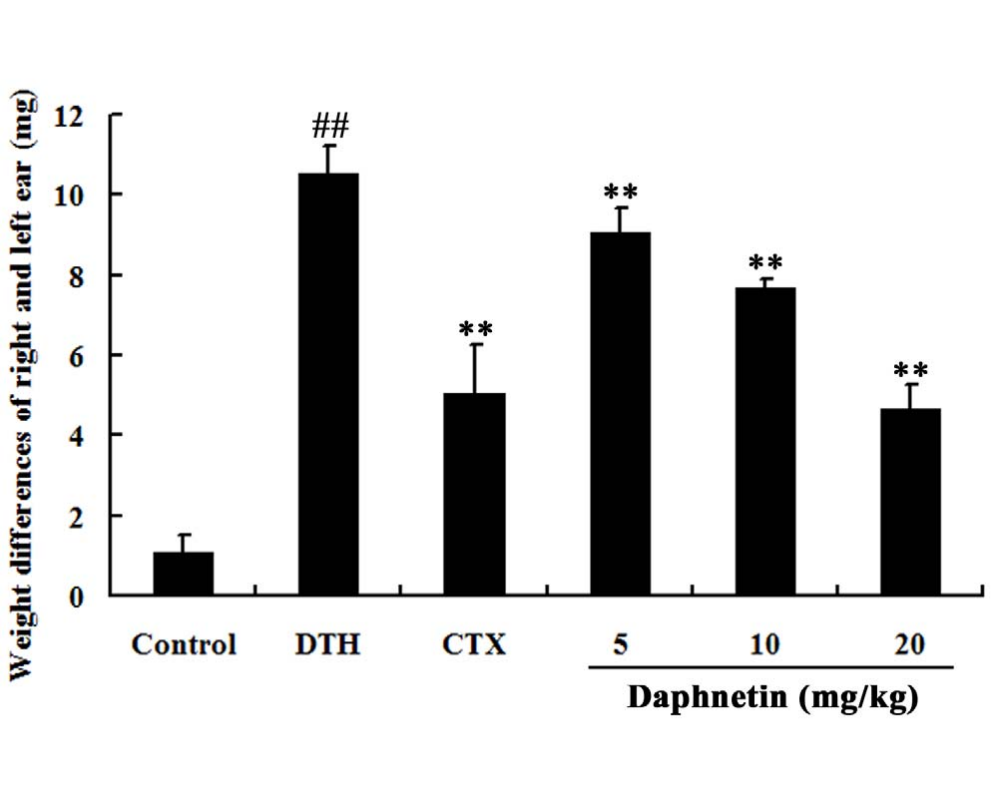
Effects of daphnetin on cell immunity of DNFB-treated mice evaluated by DTH. The DTH reaction was evaluated by the increase in the ear patch weight (8-mm punches) between the left and right ear was measured 24 h after the second challenge. ^##^ *P*<0.01 vs. Control group. **P<0.01 vs. DTH group.

**Figure 10:**
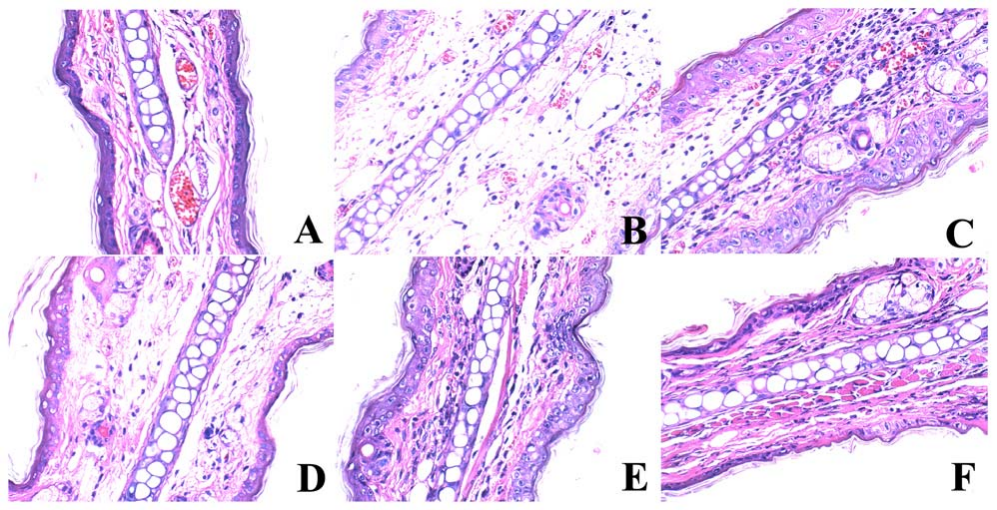
Daphnetin reduced ear swelling and leukocytes infiltration. Histological changes in right ear of mice at 24 h following elicitation with DNFB. (A) Control group: microphotograph showed normal structure of the ear; (B) DTH group: microphotograph showed histopathologic changes (edema, infiltration of inflammatory cells) of the ear; (C) CTX group; Daphnetin group (D: 5 mg/kg; E; 10 mg/kg; F: 20 mg/kg): microphotograph showing decreased histopathologic changes of the ear.

To determine the effects of daphnetin on the allergy response *in vivo*, the ear swelling reaction was evaluated in mice. As shown in Figure 9, compared with the DTH group, the ear swellings were significantly decreased in mice treated with daphnetin and standard drug CTX. Histological specimens were prepared from ear lobes obtained after 24 h of final DNFB treatment. As shown in Figure 10, no notable histological changes of the ear skin were observed, connective tissue was tightly packed and infiltration of inflammatory cells in control group. Exposure to DNFB resulted in marked increases in skin thickness and weight. Topical application of daphnetin dose dependently inhibited ear swelling in DNFB sensitized mice. The inflamed auricles of the mice were examined histologically by (H&E) staining.

## Discussion

Immunosuppressive agents are an important drug class that is valuable for the treatment of various human diseases. These drugs have been considered to be useful in controlling and treatment of autoimmune diseases. Although there are a variety of immunosuppressive drugs such as cyclosporin A (CsA), tacrolimus (FK506), Methotrexate, Azathioprine and Rituximab, the toxicity is the major obstacle in the widely use of these immunosuppressive drugs [23], [24], [25]. Therefore, new and safe immunosuppressive drugs against acute and chronic rejection are eagerly awaited. There has been an increasing interest to explore phytochemicals with therapeutic potential in alloimmune diseases in that they can be purified, synthesized, and modified in chemical structure for new drug design and often have low toxicity. In this study, we reported that a new discovered compound from Daphne Korean Nakai, daphnetin, has potent immunosuppressive activity *in vitro* and *in vivo*. Daphnetin is currently being used clinically in China to treat coagulation disorder. Intravenous injection of daphnetin at a dose of 40 or 80 mg/ kg inhibited rabbit platelet aggregation and reduced rat platelet adhesion [26]. The human thrombus was significantly inhibited when daphnetin was given orally 800 mg/ kg for 2 weeks [27]. The usual clinical dose range for daphetin was 450 mg three times a day. Therefore, in this study, we used daphnetin at concentrations of 20 mg/kg or under in vivo without the dangers of anti-coagulation.

Autoreactive T-cell proliferation has been implicated in the pathogenesis of a variety of autoimmune diseases [28]. In this study, we focused on T lymphocytes, investigated the inhibitive activity of daphnetin on ConA-induced T-cell proliferation. The results showed that daphnetin had a high inhibitory capacity for T lymphocyte proliferation with a relatively low cytotoxicity. Cellular proliferation is regulated primarily by regulation of the cell cycle, which consists of distinct sequential phases (G0/G1, S, G2). Activation of each phase is dependent on the proper progression and completion of the previous one[29]. The results of FACS indicated that the inhibitive activity of daphnetin on T lymphocytes over-proliferation may be related to its interdiction of DNA replication in the G1-S stage and regulation of cell mitosis cycle.

Cytokines are important modulators and effectors in the immune system. In particular, multiple proinflammatory cytokines have been proved to be closely associated with many autoimmune diseases. It was clear that Th1 cells produced IL-2, IFN-γ, IL-12, and other cytokines when stimulated; and Th2 cells produced IL-4, IL-5, IL-6, and IL-10 [30]. Overactivation of either pattern could lead to Th1 or Th2 polarization. The imbalance of Th1/Th2 would lead to immunological disease, such as rheumatoid arthritis, type-1 diabetes and multiple sclerosis [31]. In this study, we used ConA as T cell mitogens, and selected IL-2, IFN-γ as Th1 cytokines and IL-4, IL-6 as Th2 cytokines to test the effect of daphnetin on modulating the Th1 and Th2 cytokines. The result showed that daphnetin could effectively rectify the Th1 and Th2 polarization in mouse T lymphocytes.

Intracellular Ca^2+^ is a quintessential intracellular messenger, and many of its cellular effects are transduced by calmodulin. CaM, a major calcium receptor, is present in both cytoplasmic and nuclear compartments [32]. The calcium/CaM complex regulates several downstream targets including protein kinases and phosphatases. Calcineurin (CaN) is a serine/threonine phosphatase enzyme that is expressed in many types of tissues such as immune cells, muscle cells and neurons. It plays important roles in regulating immunological responses in lymphocytes [33]. Immunosuppressant of important components of the Ca^2+^ signaling pathway revealed that ConA-induced NFAT activation depends on a Ca^2+^/calmodulin/calcineurin pathway whereas ConA-induced NF-kB activation depends on a Ca^2+^/calmodulin/CamKII pathway that is also affected by calmodulin [34], [35]. Our results show that in ConA group, after activation by both [Ca^2+^]i and CaM, CaN dephosphorylates its cytoplasmic substrate, the nuclear factor of activated T cells (NFAT), which is a member of the family of transcription factors. Then the activated NFATc translocates from the cytosol into the nucleus. As expected, daphnetin inhibited the ConA-induced NFAT2 translocation into the nucleus and prevented T cell activation. NF-κB is also Ca^2+^-dependent transcription factor that responsible for the activation of immune response especially in T lymphocytes. A variety of different protein kinases have been implicated in the TCR-induced NF-κB activation pathway [36]. One of these is calmodulin-dependent kinase II (CaMKII), which is activated by Ca^2+^-loaded CaM. To determine whether NF-kB activation pathways require the action of CaMKII, the effects of daphnetin on the CaMKII phosphorylating activity after stimulation by ConA were examined. The results showed daphnetin clearly inhibited p-CaMKII and NF-kB.

Delayed-type hypersensitivity (DTH) reaction is usually regarded as cell-mediated immune response and plays an essential role in the immune diseases, and it is a cell-mediated pathologic response involved with T cell activation and the production of many cytokines [37], [38]. These immune diseases are usually treated by immunosuppressants, which posses a strong anti-DTH activity. In the present study, we have shown that daphnetin reduced the immune response in DTH. Ear swelling in DTH is primarily the result of *in vivo* functions of antigen specific CD4^+^ T cell response. The effect of daphnetin on DTH was further supported by histopathological analysis, which showed that DNFB-induced increase in ear thickness and infiltration of leukocytes into epidermis and dermis were suppressed by daphnetin. We also found that DNFB treatment increased the number of CD4^+^ and CD8^+^ T cells in DTH model. In contrast, daphnetin treatments reduced the CD4^+^ T and CD8^+^ T cell numbers after DNFB treatment. The ratio of CD4^+^/CD8^+^ was much higher in the model mice than that in the control mice. The ratio of CD4^+^/CD8^+^ in DTH mice serum was decreased by daphnetin treatment in dose-dependent.

In conclusion, our results demonstrated that daphnetin had an immunosuppressive activity on T-cell activation both *in vitro* and *in vivo*. The immunosuppressant effects were tightly associated with regulation of cell mitosis cycle, modulation of Th1 and Th2 cytokines *in vitro*. The inhibitory effects might be associated with NFAT and NF-KB translocation. *In vivo*, we also provide experimental evidence, for the first time, to demonstrate the protective effect of daphnetin on DTH model induced by DNFB. These findings extended our understanding of the immunomodulatory effects of daphnetin and suggested the potential of it as a new and effective phytochemical compound for the treatment of T-cell mediated immune diseases. Because daphnetin potentially have low toxicity and few side effects [39], [40], our study suggests the possibility of developing daphnetin as a novel safe immunosuppressant.
